# A new species of *Lecidea* (Lecanorales, Ascomycota) from Pakistan

**DOI:** 10.3897/mycokeys.38.26960

**Published:** 2018-08-07

**Authors:** Memoona Khan, Abdul Nasir Khalid, H. Thorsten Lumbsch

**Affiliations:** 1 Department of Botany, University of the Punjab, Lahore, Pakistan University of the Punjab Lahore Pakistan; 2 Science & Education, The Field Museum, 1400 South Lake Shore Drive, Chicago, IL 60605-2496, U.S.A. The Field Museum Chicago United States of America

**Keywords:** Asia, Lecideaceae, lichenised fungi, new species, taxonomy

## Abstract

We describe here a new species, *Lecideaaptrootii*, in *Lecidea* sensu stricto from Swat Valley, Pakistan. It is most similar to *L.fuscoatra* in having an areolate thallus and black, lecideine apothecia with a persistent margin. However, *L.aptrootii* can be readily distinguished by having smaller ascospores (average length 8-10 µm). In phylogenetic analyses, using ITS and nuLSU rDNA sequences, *L.aptrootii* forms a sister-group relationship to *L.grisella*, which differs in having a rimose thallus.

## Introduction

Pakistan is a country with a broad altitudinal range from sea level at the Arabian Sea to the second highest point of the world (K-2) at 8,611 m (Khan 1991). This variation in altitude is associated with diverse ecosystems, constituting 18 distinct ecoregions (IUCN 2006). Consequently, the lichen flora of this region is expected to be rich but so far largely unknown due to lack of detailed surveys (Aptroot and Iqbal 2012). Swat is the focal point of the Hindu Kush Himalayan region of Pakistan and its lichen flora is currently being studied by the first author (MK). The area is primarily montane and spreads over 8220 km^2^ of land with altitudinal variation ranging from 600 m in the south to more than 6000 m in northern high peaks (Ahmad et al. 2015). The known lichen flora of Swat represents roughly 26% of the total lichen flora of the country but almost all reports are from small localities and easily approachable picnic spots (Aptroot and Iqbal 2012). In our studies of the lichen flora of this region, an interesting, crustose lichen of the genus *Lecidea* was found.

The genus *Lecidea* Ach. (Lecideaceae) that was originally described by Acharius (1803), underwent many systematic changes and its traditional wide circumscription (Zahlbruckner 1926) dramatically changed over the last decades (Hafellner 1984; Hertel 1967, 1977, 2006). Of the 427 species included in *Lecidea* sensu lato (Kirk et al. 2008), only about 100 belong to *Lecidea* sensu stricto based on, amongst other characters, the presence of a *Lecidea*-type ascus (Hertel 2006). The taxonomy of *Lecidea* species is complex because of the morphological variation within and amongst species. The species circumscription in *Lecidea* needs revision and molecular data are helpful in interpreting subtle morphological differences that have been considered as intraspecific variability (Schmull et al. 2011). From Pakistan, so far five *Lecidea* spp. have been reported, viz. *L.atrobrunnea* (Ram.) Schaer., *L.atroviridis* (Arnold) Th.Fr., *L.bohlinii* H. Magn, *L.portensis* Nádv. and *L.tessellata* Flörke (Aptroot and Iqbal 2012). The new record of a saxicolous lichen in the Swat district adds a sixth species of the genus. Below, this species is described morphologically and chemically and molecular evidence identified that it represents a new taxon in *Lecidea* sensu stricto.

## Material and methods

### Morphological and chemical studies

Collections were made in August 2016 during a mycological survey of Gabin Jabba and Malam Jabba (Swat Valley) where altitude varies from 600 m to 2500 m. These areas have a moist temperate climate and remain under snow cover during winter while the summer season is accompanied by a significant amount of rainfall. Standard microscopy and spot tests (Hale 1979) were used for identification. Measurements were made from free-hand sections mounted in water. Amyloid reactions were tested with Lugol’s solution with and without pretreatment with 10% KOH. High performance thin layer chromatography (HPTLC) was performed using solvent C following standard methods (Arup et al. 1993, Lumbsch 2002, Orange et al. 2001).

### DNA extraction, PCR amplification and sequencing

We used apothecia to extract DNA with Fungal/Bacterial DNA Miniprep Kit (Zymo Research Corp., Irvine, CA) following the manufacturer’s instructions. Molecular data were generated for two loci: the internal transcribed spacer (ITS) and the nuclear large subunit (nuLSU) ribosomal DNA. The primer pair ITS1F (Gardes and Bruns 1993) and ITS4 (White et al. 1990) were used to amplify the ITS region; and primer pair AL2R (Mangold et al. 2008) and LR6 (Vilgalys and Hester 1990) were used to amplify the nuLSU region. Polymerase chain reactions (PCR) were performed in 12.5 µl volume per reaction using MyTaq^TM^ Red Mix (Bioline International, Toronto, Canada). PCR protocol for the ITS region consisted of initial denaturation of 5 min at 94 °C, 40 cycles of 30 sec at 94 °C, 30 sec at 48 °C, 1.5 min at 72 °C and a final extension of 5 min at 72 °C. PCR protocol for LSU consisted of initial denaturation of 75 sec at 94.5 °C, 35 cycles of 35 sec at 95 °C, 55 sec at 55 °C, 42 sec at 72 °C and a final extension of 10 min at 72 °C. PCR products were visualised on 1% agarose gel and cleaned using Exo SAP-IT (USB, Cleveland, Ohio, USA) following the manufacturer’s instructions. Cycle sequencing reactions were performed using BigDye Terminator v3.1 (Applied Biosystems, Foster City, CA, USA) with the same primers as used for the PCR amplification. The sequencing reactions were run on an ABI 3730 48-capillary electrophoresis DNA analyser sequencer according to established protocols (Applied Biosystems) at the Pritzker Laboratory for Molecular Systematics at the Field Museum, Chicago, IL, USA.

### Phylogenetic analyses

The ITS locus for two specimens and nuLSU gene for one specimen were successfully amplified and sequenced. Sequences of other *Lecidea* spp. based on initial BLAST searches and those used in a recent study on *Lecidea* by McCune et al. (2017) were used in phylogenetic analyses (Table 1). *Bellemereacinereorufescens* (Ach.) Clauzade & Roux was used as the outgroup. Multiple sequence alignments for each individual locus were performed using programme MAFFT v7 with all parameters set to default values (Kotah and Standley 2013). The ends of alignments were trimmed to nearly an equal number of sites for all sequences. All gaps were treated as missing data. ITS and nuLSU sequences were concatenated in Bioedit v7.2.5 (Hall 1999) using the append file option. Maximum likelihood analyses were performed via CIPRES Science Gateway (Miller et al. 2010) employing RAxML-HPC2 on XSEDE (Stamatakis 2014). For bootstrapping, the GTRCAT model was selected. One thousand rapid bootstrap replicates were run. Two molecular analyses (one with ITS and another with combined ITS & nuLSU rDNA) were performed since only a limited amount of nuLSU data were available, whereas a larger number of ITS sequences, available in GenBank, allow for in-depth inference of the phylogenetic position of the new species.

**Table 1.** GenBank accession numbers of sequences used in phylogenetic analyses.

Species	ITS	nuLSU
* Bellemerea cinereorufescens *	KY800500	-
*Lecideaandersonii* 1	EU257685	-
*Lecideaandersonii* 2	EU257683	-
*Lecideaandersonii* 3	EU257684	-
***Lecideaaptrootii* 1** Gabin Jabba (GB-1)	**MH618901**	-
***Lecideaaptrootii* 2**** Malam Jabba (MJ-3)	**MH594348**	**MH594349**
*Lecideaatrobrunnea* 1	EU259897	HQ660535
*Lecideaatrobrunnea* 2	EU259898	AY532993
*Lecideacancriformis* 1	EU357674	-
*Lecideacancriformis* 2	EU257671	-
*Lecideacancriformis* 3	EU257672	-
*Lecideafuscoatra* 1	HQ605929	HQ660541
*Lecideafuscoatra* 2	HQ605926	AY756339
*Lecideafuscoatravargrisella* 1	HQ605931	HQ660542
*Lecideafuscoatravargrisella* 2	HQ605928	-
*Lecidealaboriosa* 1	EU259902	KJ766586
*Lecidealaboriosa* 2	EU259901	DQ986882

## Results and discussion

### Taxonomy

#### Lecidea aptrootii

Taxon classificationFungiLecidealesLecideaceae

M. Khan, A.N. Khalid, H. T. Lumbsch sp. nov.

825562

Figures 3 , 4

##### Type.

PAKISTAN. Khyber Pakhtunkhwa Province, Swat district, Gabin Jabba valley, 1600 m alt., 37.1706°N, 72.3711°E, 18 Aug 2016, AN Khalid, GB-1 (Holotype LAH-35505).

##### Diagnosis.

Saxicolous, thallus irregularly areolate, apothecia epruinose, lecideine with persistent margin, asci with tholus, I+ blue, ascospores simple, ellipsoid with average size of 8–10 × 4.5–5.5 µm.

##### Description.

Thallus crustose, irregularly areolate, subcontiguous; prothallus usually indistinct, black when present; areoles flat, up to 1.2 mm in diameter and 300 µm thick, uniformity in colour from centre to edge; surface rough, not shiny, greenish-grey to light brown; Cortex not clearly differentiated, up to 31.5 μm in thickness; medulla I-, medullary hyphae thin walled, compactly arranged, 2.4–3.2 µm in diameter; photobiont layer up to 63 µm thick, algal cells 12.8–14.4 µm; apothecia black, round to irregular in outline, up to 1.5 mm in diameter, lecideine, epruinose with slightly raised, black, thin and persistent margin, frequently present, disc black, flat to slightly convex, proper exciple thin, dark brown to black; epihymenium light brown to dark brown, 8–16 µm; hymenium hyaline to olivaceous brown, (60)-70–98 µm tall; subhymenium light brown to dark brown, Hypothecium darkly pigmented throughout, Asci clavate with distinct tholus, the tip I+ blue, 8-spored, 50–68 × 12.30–16.70 µm; Ascospores simple, ellipsoid, (7)8–10(11) × (4)4.5–5.5(6) µm; paraphyses branched and net-like, 1.6–2.4 µm wide, not expanded at tips; vegetative propagules and conidiomata not seen.

##### Chemistry.

Thallus K-, KC+, C+ Red, P-, UV-. Gyrophoric acid, schizopeltic acid and 2’-*O*-methylperlatolic acid were detected with HPTLC.

##### Distribution and ecology.

The species is so far only known from two collections in the Swat district in Pakistan, where the species occurs on exposed siliceous rocks between 1600 and 1900 m altitude.

##### Etymology.

The epithet “aptrootii” refers to the lichenologist André Aptroot who has contributed to the knowledge of lichen diversity in Pakistan (e.g. Aptroot and Iqbal 2012) and has indicated to the first author that the material from Pakistan might represent an undescribed species.

##### Notes.

*Lecideaaptrootii* belongs to *Lecidea* sensu stricto (Hertel 2006). The new species is a member of Lecideasubgen.Lecidea, according to the generic sub-classification as suggested by Rambold (1989). In the field, it looks like *L.fuscoatra* with an areolate thallus and black apothecia. A microscopic study revealed it differs from that species in having smaller ascospores. Another similar species in the complex is *L.grisella*, which, however, is readily distinguished by having a rimose rather than areolate thallus (Aptroot and Van Herk 2007). Recently, *Lecideagrisella* has been reported from China, which might belong to *L.aptrootii* and has ascospores 8-12(13) µm in length (Zhao et al. 2017) that overlap with an average spore length for *L.aptrootii* i.e. 8-10 µm. Additional studies are necessary to determine whether the Chinese material belongs to *L.aptrootii* or represents an additional lineage in this complex. Molecular data in *Lecidea* are helpful to interpret morphological features previously considered as intraspecific variation (Schmull 2011).

**Figure 1.**
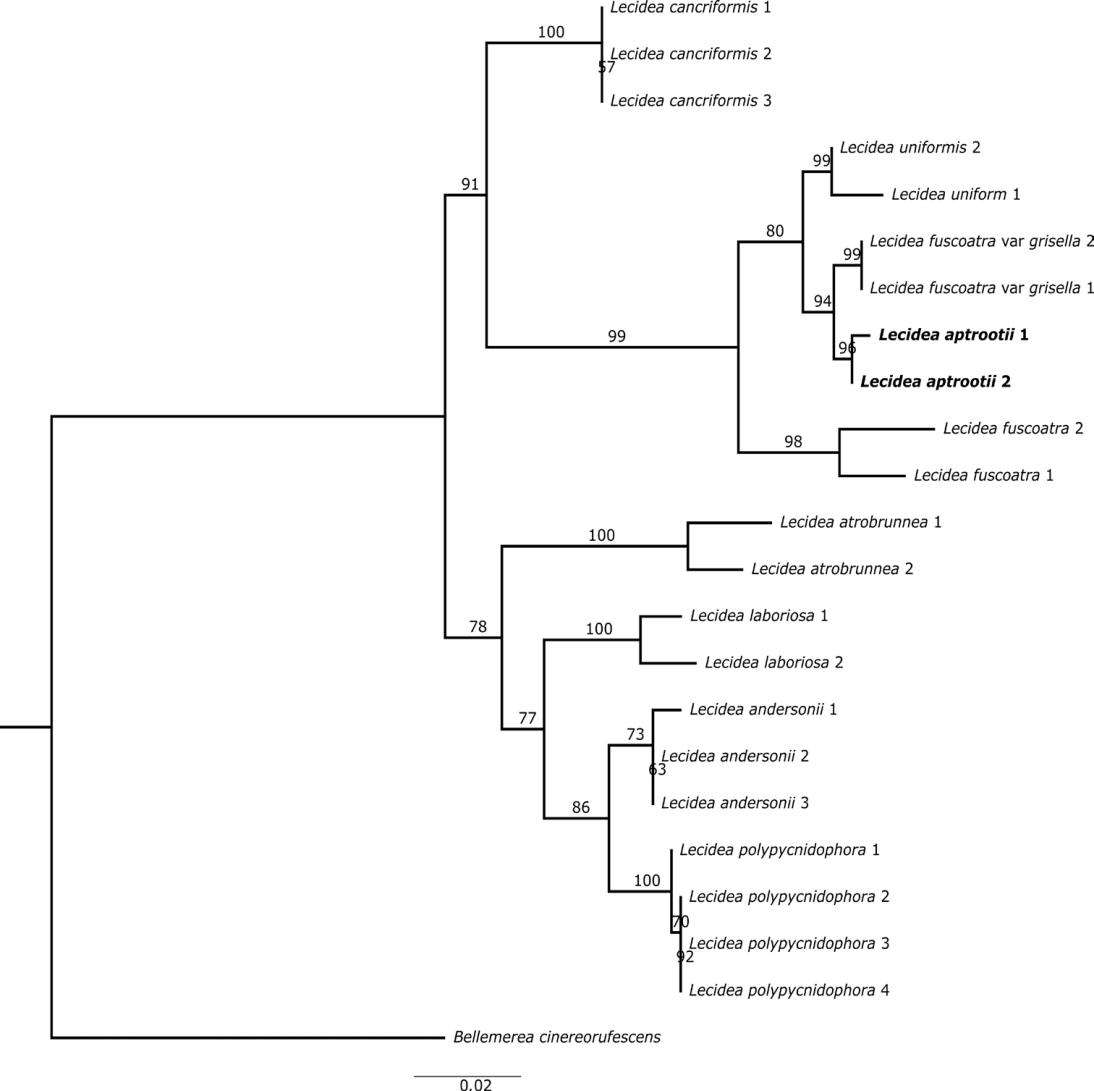
Most likely phylogenetic relationship of *Lecideaaptrootii* and associated taxa inferred with ITS data based on rooting with *Bellemereacinereorufescens* as outgroup. Branch lengths are based on the estimated number of substitutions per site.

**Figure 2.**
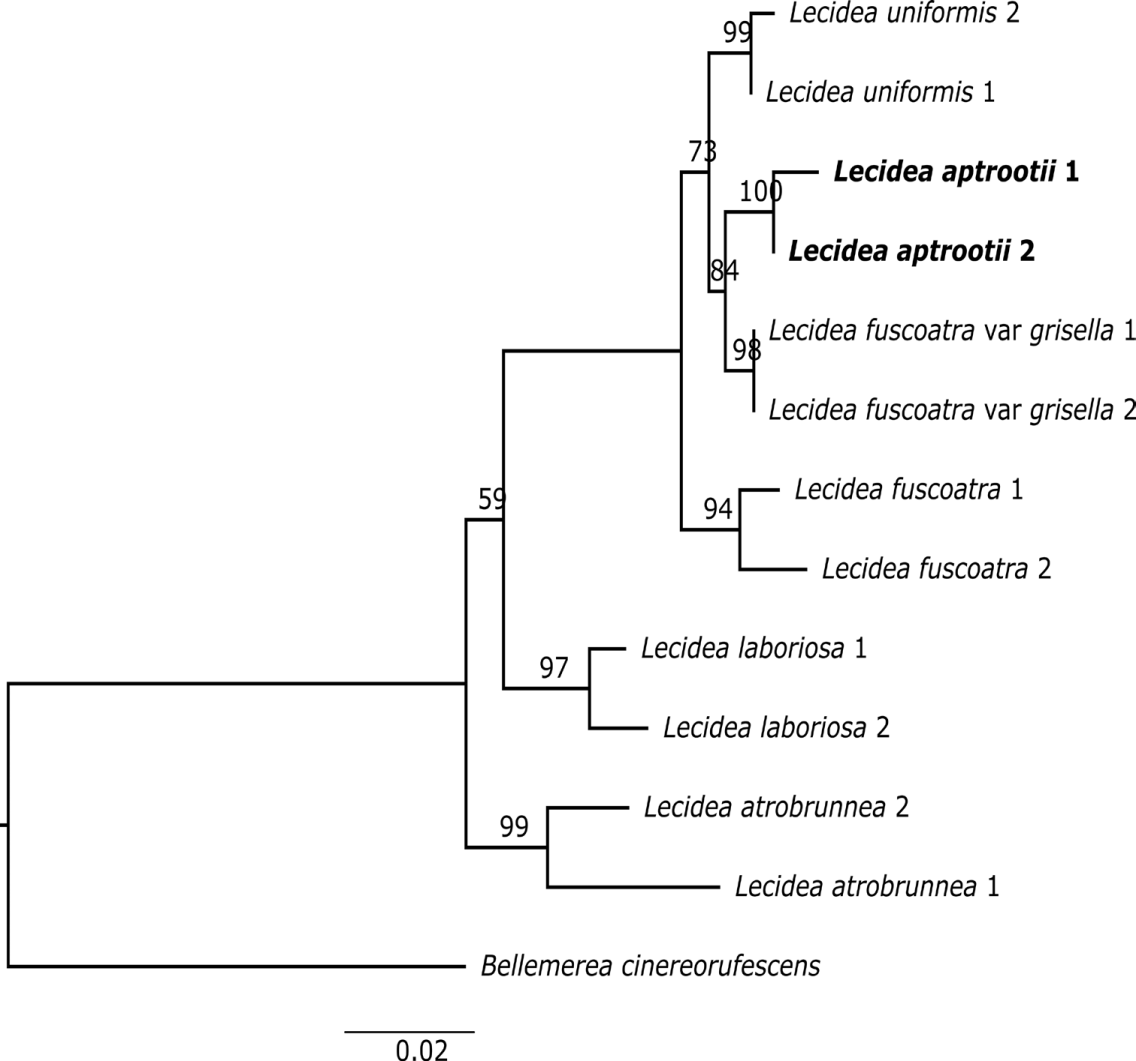
Phylogenetic relationships of *Lecideaaptrootii* and associated taxa inferred from ITS & nuLSU rDNA data under maximum likelihood. *Bellemereacinereorufescens* was used as outgroup. Bootstrap values indicated at nodes.

The areoles of *L.fuscoatra* have a differentiated black or grey margin, in contrast to the black cortex, whereas in *L.aptrootii*, the margins of areoles are concolorous with the areoles. This is a common feature of *L.aptrootii* and the recently described *L.uniformis* from North America (McCune et al. 2017). However, the two species differ in the branching of paraphyses and presence of tholus in the asci. Further, molecular data support that they represent distinct lineages (Figs 1–2).

In addition, *L.oreophila* K. Knudsen & Kocourk. with irregularly areolate thallus, light to dark grey upper surface and epruinose apothecia, might be confused with *L.aptrootii* but the former has apothecia that are usually higher than areoles and rarely branched paraphyses with expanded apices up to 5 µm (Knudsen and Kocourková 2014).

Molecular analyses of ITS and two ribosomal loci (ITS & nuLSU) dataset (605 and 1433 unambiguously aligned positions in ITS and two loci dataset, respectively) support the fact that the Pakistan collections are phylogenetically distinct from the morphologically similar *L.fuscoatra* and *L.uniformis* (Figs 1–2). In fact, the morphologically different *L.grisella* forms a well-supported sister-group relationship with *L.aptrootii*.

##### Additional specimen examined.

Pakistan, Khyber Pakhtunkhwa province, Swat district, Malam Jabba valley, 1900 m alt., on rock, 20 Aug 2016, AN Khalid, MJ-3 (LAH-35506).

**Figure 3.**
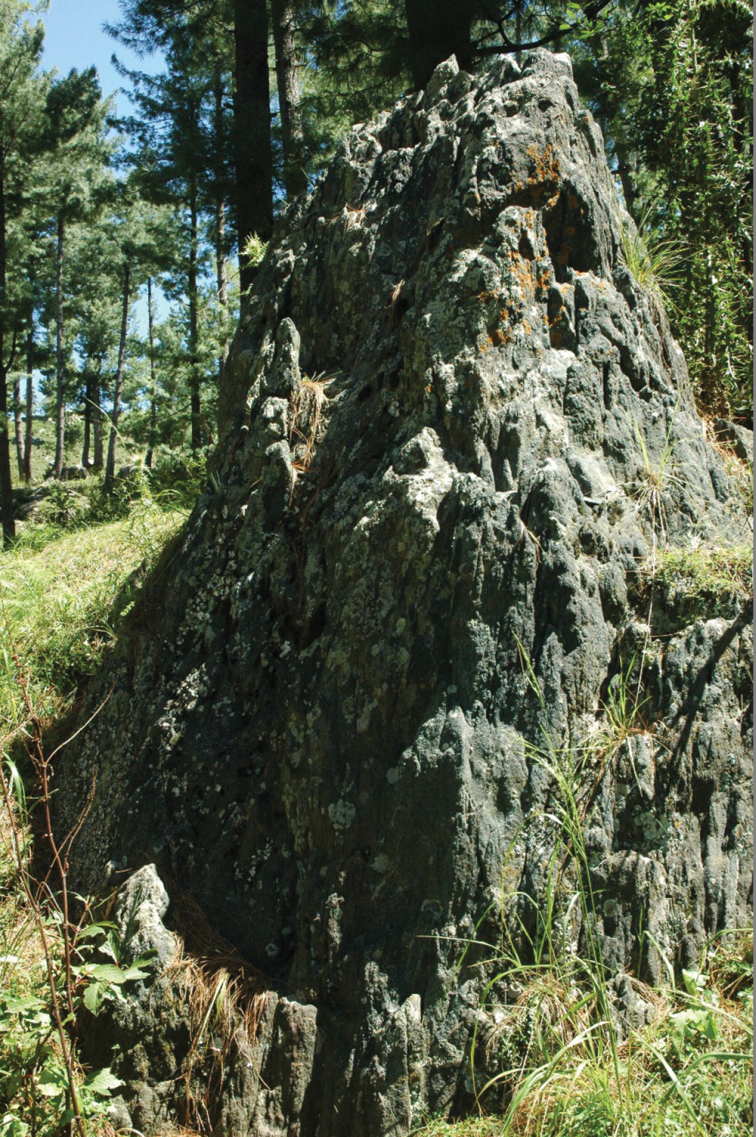
*Lecideaaptrootii* in natural habitat.

**Figure 4.**
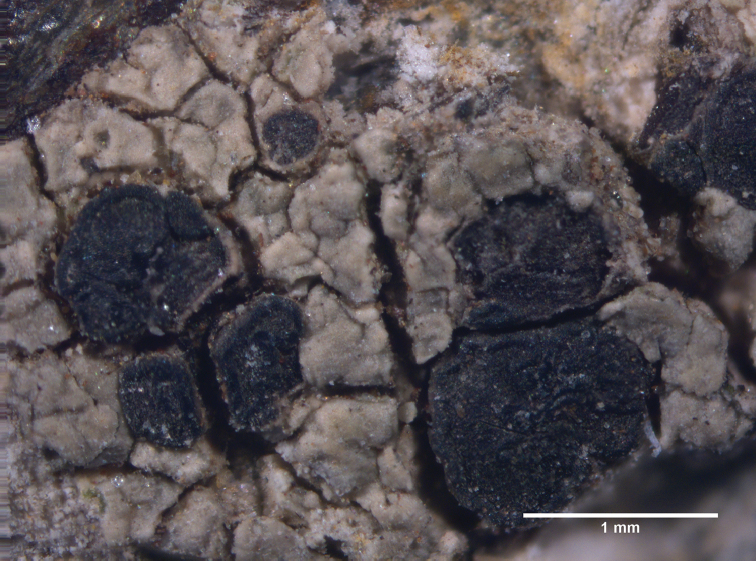
*Lecideaaptrootii*. Thallus and apothecia of the holotype.

## Supplementary Material

XML Treatment for Lecidea aptrootii
